# Do Biopesticides Affect the Demographic Traits of a Parasitoid Wasp and Its Biocontrol Services through Sublethal Effects?

**DOI:** 10.1371/journal.pone.0076548

**Published:** 2013-09-30

**Authors:** Antonio Biondi, Lucia Zappalà, John D. Stark, Nicolas Desneux

**Affiliations:** 1 Department of Agri-food and Environmental Systems Management, University of Catania, Catania, Italy; 2 French National Institute for Agricultural Research (INRA), Sophia-Antipolis, France; 3 Departments of Entomology-Puyallup Research and Extension Center, Washington State University, Puyallup, Washington, United States of America; Texas Tech University, United States of America

## Abstract

Pesticide risk assessments are usually based on short-term acute toxicity tests, while longer-term population dynamic related traits, critical to the success of biological control and Integrated Pest Management (IPM) programs, are often overlooked. This is increasingly important with respect to new biopesticides that frequently cause no short-term acute effects, but that can induce multiple physiological and behavioral sublethal effects, leading to a decrease in population growth and ecosystem services. In this study we assessed the lethal and sublethal effects of six biopesticides [abamectin, azadirachtin, *Bacillus thuringiensis*, borax plus citrus oil (Prev-Am®), emamectin benzoate, and spinosad], used in tomato crops to control the invasive pest *Tuta absoluta* (Lepidoptera: Gelechiidae), on adults and pupae of the parasitoid *Bracon nigricans* (Hymenoptera: Braconidae). Data on female survival and production of female offspring were used to calculate population growth indexes as a measure of population recovery after pesticide exposure. Spinosad caused 100% and 80% mortality in exposed adults (even 10 d after the treatment) and pupae, respectively. Although most of the biopesticides had low levels of acute toxicity, multiple sublethal effects were observed. The biocontrol activity of both females that survived 1-h and 10-d old residues, and females that emerged from topically treated pupae was significantly affected by the application of the neurotoxic insecticides emamectin benzoate and abamectin. Furthermore, very low *B. nigricans* demographic growth indices were estimated for these two insecticides, indicating potential local extinction of the wasp populations. Among the tested products, *Bt* proved to be the safest for *B. nigricans* adults and pupae. Our findings emphasize that acute toxicity assessment alone cannot fully predict the actual impact of pesticides on non-target parasitoids. Thus, sublethal effects related to the species specific life-history variables must be carefully considered in order to assess pesticide risks and to incorporate new pesticides, including biopesticides, into IPM programmes.

## Introduction

The effects of pesticides and other toxicants on organisms have been traditionally assessed using simplistic measures of the acute mortality, or the evaluation of mortality induced by field recommended doses [1], [2]. The lethal concentration estimate, e.g. LD_50_ or LC_50_, is a simple approach that enables a rapid evaluation and comparison of the effect of several toxicants on the individuals of a given species [3]–[6]. A major criticism of the LC_50_ as a measure of effect in target and non-target organisms is that this estimate does not take into account the total effect on population dynamics. The projection of the actual toxic effects on the population level is further complicated by the fact that they may result from both individuals dying, and from impaired surviving individuals due to sublethal effects. The latter are defined as physiological and behavioral effects on individuals that survive exposure to a toxicant (see Desneux et al. [2] for a thorough review).

Sublethal effects may impair various physiological and behavioral traits on the exposed organisms (e.g. reproductive [7]–[10], longevity [11]–[13], orientation [14]–[17] and feeding behaviors [18], [19]). The ability of pesticide-treated individuals that survive exposure to provide ecosystem services, such as predation and parasitization, can be strongly compromised [20]–[22]. Moreover, some species can suffer high levels of mortality and recover quickly because they have high population growth rates, short generation times and/or an early onset of reproductive activity [23]. By contrast, other species may become locally extinct after exposure to a toxicant at a concentration that does not kill all individuals because sublethal effects severely impact surviving individual reproductive capacity [2], [24]. To determine the total effects of pesticides, life table data can be developed in demographic studies and used in mathematical models to predict (ordinary or partial differential *equation models*) or project (*matrix models*) population dynamics 23,25.

Demographic toxicological analysis, estimating the total effect of insecticides on populations, is increasingly important when choosing new pesticides for Integrated Pest Management (IPM) purposes [26]. Among newly developed pesticides, biopesticides are a wide range of agrochemicals composed of, or derived from, organisms (such as microbes, nematodes, and botanicals), or by natural occurring materials (mostly minerals) [27], [28]. In the past decades, these pesticides have been more frequently considered for the development of environmentally-sound and IPM strategies [29]–[31]. Their use mainly aims at reducing the non-target effects that come with conventional pesticides. However, the origin of a given compound does not necessarily relate to the toxicological properties, or the ramifications of the effects of its physical and chemical characteristics on an arthropods physiology and behavior [32].

The objective of the present study is to provide an estimation of lethal and sublethal toxicity of six newly developed biopesticides on a parasitoid wasp. We focused on a new plant/pest/natural enemy association model, the invasive South American tomato pest *Tuta absoluta* (Meyrick) (Lepidoptera: Gelechiidae) and one of its natural enemies, the European ectoparasitoid *Bracon nigricans* Szépligeti (Hymenoptera: Braconidae). *Bracon nigricans* is widespread in the Palaearctic area and it attacks various Lepidopteran species [33], including *T. absoluta* in Europe [34], [35]. Parasitoids are organisms that have evolved a wide spectrum of fine and specialized physiological and behavioral mechanisms to locate, attack, and develop on their hosts 36–40. Braconid wasps are key natural enemies of several insect pests within a broad range of cropping systems worldwide [34], [41]. In effect, the development of their populations and consequently of their ecosystem services can be disrupted by most crop protection practices that include applications of broad spectrum insecticides [1], [2]. The arrival of *T. absoluta* in the western Palaearctic area has caused yield losses in tomato crops and as a result, the use of insecticides has increased owing to this pest [42]–[44]. Thus, understanding the risks posed by these insecticides is crucial for developing long-term sustainable pest control tactics, such as IPM and organic control approaches against exotic pests. We assessed the effects of field concentrations of the six biopesticides when adults were exposed to dry residues (1-h and 10-d old) and also when parasitoid cocooned pupae were directly sprayed with the pesticides. We used the results to calculate demographical indexes as alternative endpoints for risk assessment purposes.

## Materials and Methods

### Insects

A *B. nigricans* colony was established by collecting individuals from infested samples during a survey of indigenous natural enemies attacking *T. absoluta* from various tomato crop sites in Southern Italy [34]. The colony was maintained in a laboratory for several generations using tomato plants infested with late-instar *T. absoluta* as hosts. The host colony was established from *T. absoluta* infested leaves collected from greenhouse tomato crops, and was thereafter maintained on tomato plants (cv. Marmande) in the laboratory. Seedling host plants were grown in small pots (0.3 L), watered, and fertilized following routine practices. Pesticide applications were strictly avoided. Tomato plants infested by mature moth larvae (3^rd^ and 4^th^ instars) were produced by releasing forty *T. absoluta* adults (1∶1 sex ratio) on 10 tomato plants inside 50×60×60 cm cages covered with a fine polyester mesh. 14±2 d after releasing the adult moths, at which point the majority of the larvae had reached the 3th instar, the plants were transferred into parasitoid rearing cages. These measured 40×40×55 cm and were built from plastic boxes with an opening that was covered by a fine mesh net. Every 3 d the rearing cages were supplied with *Tuta absoluta*-infested plants and honey droplets.

Newly emerged 0/48-h old adults and 5/6-d old cocooned pupae (i.e. mature pupae, since the entire pupal development lasts about 7 d [35]) were contained in 150 mm Petri dishes ventilated by a 4 cm^2^ opening covered with a fine mesh net. Two females and four males were released into this arena, which contained excised *T.absoluta* infested tomato leaves. To mimic the field scenario in which parasitoid wasps use external nutrient inputs for adult feeding [45], [46], honey droplets were provided in the arena daily. After 2 d of parasitisation activity, the wasps were removed and the infested and parasitized material was monitored in a climatic cabinet. The leaves bearing cocooned pupae and newly emerged adults were collected for bioassays 8 and 11 d later, respectively.

The growing host plants and the two insect rearings were held in three growing chambers (26±1°C; 60±10% RH; 14∶10 L.D.); while the bioassays were conducted in a climatic cabinet maintained at the same environmental conditions as the growing chambers.

### Insecticides and treatments

The tested pesticides were six bioinsecticides marketed for the control of various pests (including lepidopterans) in several cropping systems, both for organic and conventional farming (Table 1). These biopesticides have been increasingly used in tomato crops owing to the recent invasion of *T. absoluta* in the Mediterranean basin [43], [47]–[50]. Four insecticides were evaluated that are active upon ingestion: the microbial *Bacillus thuringiensis* Berliner var. Kurstaki (*Bt*), the plant-derived azadirachtin (neem oil extract preparation) and two semi-synthetic microbial-derived products, abamectin and emamectin benzoate (hereafter named emamectin). One non-conventional pesticide that is active when in contact with the pest (a mix of citrus essential oils and borax salt; hereafter named BCO) was also included in the study, as well as a biopesticide active both when in contact and when ingested, the bacterial metabolite-derivate spinosad. Considering spinosad has marked acute toxicity toward many hymenopteran parasitoid species [32], it was used as a positive control for lethal exposure to toxicants to ensure that our exposure bioassay was reliable (i.e. wasps exposed to dried insecticide residues were exposed effectively to toxicants). The highest recommended rates for tomato crops were used in our experiments. Detailed information on the commercial products is provided in Table 1.

**Table 1:** Tested biopesticides.

Active ingredient	Trade name	Field rate (a.i.%)	Chemical family	Mode of action	Crops	Target pests
Abamectin	Cal-EX EW®	75 ml hl^−1^ (1.8)	Avermectin	Ingestion. Chloride channel activator	Tomato, eggplant, sweet pepper, strawberry, lettuce, cucumber, melon, cabbages, citrus, grape, ornamental plants and flowers, forest trees	Mites. thrips, psyllids, aphids, leafminers, moths
* Azadirachtin A+B	Oikos®	150 ml hl^−1^ (3.2)	Botanical	Ingestion. Moulting disruptor	Tomato, eggplant, sweet pepper, strawberry, carrot, fennel, beans, cabbages, cucurbit crops, garlic, onion, leek, leafy vegetables, celery, stone fruits, pome fruits, actinidia, walnut, chestnut	Thrips, Hemiptera, Lepidoptera
* *Bacillus thuringiensis* var. *kurstaki* strain SA12	Costar® WG	200 g hl^−1^ (90000 I.U. µg^−1^)	Cry proteins	Ingestion. Disruptor of insect midgut epithelium	Tomato, eggplant, sweet pepper, strawberry, artichoke, corn, cotton, tobacco, potato, leafy vegetables, cucurbits, sugar beet, cabbages, sugar beet, beans, soybean, sunflower, citrus, grape, olive, actinidia, chestnut, ornamental plants, forest trees	Lepidoptera
Emamectin benzoate	Affirm®	150 g hl^−1^ (0.95)	Avermectin	Ingestion. Chloride channel activator	Tomato, eggplant, sweet pepper, strawberry, beans, artichoke, lettuce, stone fruits, pome fruits, grape, cole crops	Lepidoptera
* Borax and citrus oil	Prev-Am®	400 ml hl^−1^ (6)	Borates tetra sodium salts and oil – essential	Contact. Miscellaneous non-specific inhibitor	Tomato, strawberry, grape	Mites, whiteflies, mealybugs, Tomato borer
* Spinosad	Laser®	75 ml hl^−1^ (48)	Spinosyn	Ingestion and contact. Nicotinic acetylcholine receptor agonist	Tomato, eggplant, sweet pepper, strawberry, potato, fennel, legumes, garlic, onion, leek, stone fruits, cucurbit crops, artichoke, leafy vegetables, caper, pome fruits, stone fruits, grape, small fruits, tree nuts, ornamental plants, grass	Thrips, Planthoppers, Lepidoptera, Coleoptera, Diptera

*Pesticides authorized also in organic farming.

Plants and cocooned pupae were sprayed with the formulated product which had been diluted with tap water and applied at the solution rate of 1000 L ha^−1^. The treatments were applied using a 2 L aerosol hand sprayer (Matabi®, Antzuola, Guipuzcoa, Spain) and the nozzle of the sprayer was directed 0.5 m towards the plants and the cocooned pupae, thus wetting them uniformly until run off. All the insecticides were stored and applied according to their label guidelines. As a result, in the case of *Bt* and azadirachtin, an acid fertilizer (Fertacid®, Bio-Intrachem Italia) was added to modify the pH to 4.5.

### Toxicity trials

#### Residual exposure of adults and assessment of insecticide persistence

The bioassays were performed by exposing newly emerged (0–24-h old) *B. nigricans* adults to dried pesticide residues on tomato leaves for three d. The treated plants were 40 cm high, 40-d old tomato plants (var. Marmande), grown from seeds in 2-L pots. Five plants for each trial were sprayed with the pesticide solution, or with tap water for the untreated controls. The plants were left to allow the pesticides to dry for 1 hour or, in order to assess the pesticide persistence, for 10 d. To allow the pesticides to age in ordinary protected cropping conditions, all the plants (for the two different delays after application of a given pesticide) were sprayed at the same time and were kept inside insect-proof cages in greenhouse conditions (minimum < *mean temperature* < maximum: 15.6°C<*25.07°C*<40°C; minimum < *mean RH* < maximum, 21%<*62.5%*<89%). Afterwards, the top-half of the plant (about 17 cm) was removed and placed into a bioassay isolator made up of two superposed plastic cups, following the experimental design proposed by Biondi et al. [48]. Five adult females and five adult males of *B. nigricans* were introduced into the described arena. At the beginning of the experiment, untreated droplets of honey were provided as food on the walls of the experimental arena. Five replicates (5×10 = 50 wasps) were performed per pesticide and for each subsequent trial. Insect survival and the number of dead parasitoids were recorded daily during the toxicity trial; parasitoids were considered dead when they remained immobile after being touched with a fine paintbrush. Surviving adults were collected for the sublethal effects assessment.

#### Topical exposure of cocooned pupae

When studying the impact of the various tested pesticides on cocooned pupae, a realistic spray exposure method was used. Tomato leaves bearing exteriorly 4–5-d old *B. nigricans* cocooned pupae were carefully attached, in groups of three specimens, onto rectangular glass plates with double-sided sticky tape according to Desneux et al. [51]. The glass plates with leaves were then treated with the pesticide solutions with the same methods described above, and with tap water for the controls. Two hours after the insecticide application, treated cocooned pupae were removed from the leaves and placed individually into glass tubes and kept in a climatic chamber (26±1°C; 60±10% RH; 14∶10 L.D.). To assess the emergence rates of each treated group of cocooned pupae, the tubes were observed twice per d for the following four d after the treatment, and then the rate of emergence was calculated for each group. A total of 423 pupae were used in the experiment (15–22 groups of cocooned pupae, i.e. 45–66 pupae, for each insecticide and the untreated control). The emerging adults were used for the sublethal effects assessment the d after their emergence, whereas the unemerged cocoons, i.e. those lacking an emergence hole, were opened in order to determine the sex of the dead pupae.

#### Sublethal effect assessment

In order to assess the potential sublethal effects of the tested biopesticides (except spinosad which caused very high levels of mortality, see the *3.1.* section) on both the surviving adults from the three-d exposure on leaves, and on the adults that emerged after the topical treatment of cocooned pupae (0–1 d after their emergence), five *T. absoluta* mature larvae and honey droplets were offered daily per each tested couple, in aerated plastic Petri dishes (130 mm diameter, 18 mm height), for three d (i.e. a total of 15 hosts and three boxes were used per each tested female). Three couples that survived the insecticide residual contact per each replicate performed for the lethal effect assessment, i.e. 3×5 = 15 couples, and twelve couples emerging from twelve groups of topically treated pupae were tested per each insecticide and trial performed.

Daily checks were performed in order to assess: (i) the number of killed larvae, (ii) the number of parasitized larvae, (iii) the number of eggs laid and (iv) the parasitoid survival. Surviving individuals were then transferred to a new box with new hosts and after the final evaluation d (the third one) they were each placed individually into glass tubes (length: 18 cm; diameter: 1.2 cm), with access to food (honey droplets, renewed weekly), under controlled environmental conditions (26±1°C; 60±10% RH; 14∶10 L.D.). Parasitoid mortality in the tubes was observed daily. The boxes bearing the larvae, both alive and parasitized, were maintained for fifteen d under the previously described climatic conditions, after which the number and the sex of the emerged progeny were recorded.

### Data analyses

Row datasets were tested for normality and homogeneity of variance using the Kolmogorov-Smirnov D test and the Cochran test, respectively, and were transformed when necessary. In particular, the percentages of surviving specimens and of emerged adults were subjected to arcsin square root transformation and the data on fertility and longevity to log (x+1) transformation. General linear model (GLM) full factorial multivariate analysis (Multivariate and Between-subjects tests) was performed on the data obtained exposing the adults to test the effects of: (i) the insecticides (*insecticide* factor), (ii) the parasitoid sex (*sex* factor), (iii) the application timing (*residue age* factor) and, (iv) the interaction of the *insecticide* factor with all the others ones on the survival of the exposed adults (lethal effect), as well as on longevity of the surviving individuals. The same procedure with the exclusion of the *sex* factor was performed to test the effects on the number of killed hosts (paralyzed and/or parasitized hosts), on the number of progeny produced and on its sex-ratio. The latter was expressed as the ratio between the males and the total progeny. To analyze the effects of the insecticides on all the investigated traits (lethal and sublethal ones) and within each trial, i.e. for adult exposure to 1-h and 10-d residues, and for topical treatment of cocooned pupae bioassays, we used an ANOVA with Tukey HSD post-hoc-analysis.

In addition, in order to provide a single result including both lethal and sublethal effects on fertility and on progeny sex-ratio, a *Reduction coefficient E*_x_ was calculated according to Biondi et al. [48] for each tested pesticide and trial. To obtain these indices, female corrected mortality (*E_mx_*) (% of mortality after the exposure to the pesticide residues and emergence% from treated pupae) and the percentages of reduction of female offspring (*E_fx_*) (calculated from the number of females produced per female during the three d of sublethal effects evaluation) were included in the formula. After that, the obtained *E_x_* values were ranked according to the four categories of the International Organization for Biological Control (IOBC) for pesticide risk assessments conducted in the laboratory: (1) harmless, (2) slightly harmful, (3) moderately harmful and (4) harmful corresponding to reductions below 30%, between 31% and 79%, between 80% and 99% and higher than 99%, respectively [3].

As the final endpoint of the study, two demographic growth indices were estimated for the female wasps exposed to 1-h old and 10-d old insecticide residues and for those emerged from topically treated pupae: (i) the *Doubling time* (*DT*), which is the time required for a given population to grow exponentially without limit to double in size and (ii) the *Intrinsic rate of increase* (*r_m_*) representing the number of times the population will multiply itself per unit of time [23]. To obtain these values, we incorporated *E_mx_* and *E_fx_* in an *equation model* containing the *B. nigricans* life table data, namely age-specific female survival and female offspring production [23], [26]. Life history data of a control population of *B. nigricans* parasitizing *T. absoluta* in similar experimental conditions, i.e. environment and host density, were previously developed by Biondi et al. [35].

## Results

### Lethal effects

**Figure 1:**
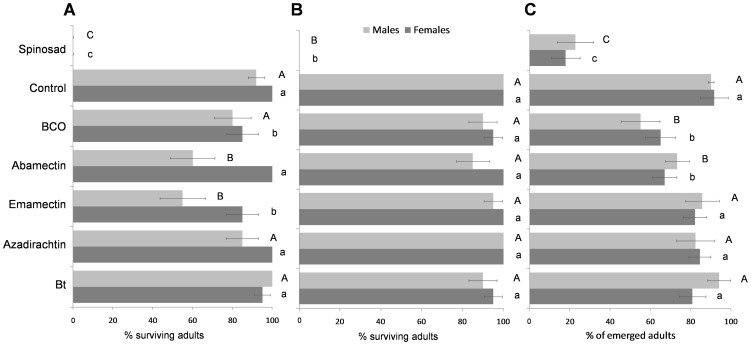
Lethal effects. Mean percentages (± SEM) of survival of *Bracon nigricans* adults when exposed to 1-h (A) or to 10-d old pesticide residues (B). Mean percentages (± SEM) of *B. nigricans* emergences from treated cocooned pupae (C). For each figure, the bars followed by the same letter (lower case letters: female; upper case letters: male) are not significantly different (*P*>0.05; ANOVA with Tukey HSD post hoc test for multiple comparisons).

Parasitoid mortality during the insecticide-exposure varied significantly among the tested biopesticides (significant *insecticide* factor) and in function of the delay between exposure to pesticides and application time (significant *residue age* factor). Moreover, the interaction of these two factors was significant (Table 2). Spinosad acute toxicity did not decrease after 10-d of the treatment and all the tested specimens died during the exposure to 1-h and 10-d old residues (Fig. 1a and 1b). Among all the other tested pesticides in the 1-h old residue trial, only BCO and emamectin (albeit only 15%) caused significant mortality of females (*F*_6,28_ = 56.513, *P*<0.001). In the same trial, emamectin and abamectin significantly reduced male survival (*F*_6,28_ = 20.542, *P*<0.001) (Fig. 1a). In the 10-d old residue trial only spinosad caused significant mortality killing all the tested females (*F*_6,28_ = 194.117, *P*<0.001) and males (*F*_6,28_ = 34.000, *P*<0.001) (Fig. 1b). The two sexes were equally susceptible to the agrochemicals, since *sex* as well as the *insecticide* × *sex* factors did not significantly affect the survival rates (Table 2). An overall lower survival rate was recorded for treated cocooned pupae rather than for exposed adults (Fig. 1). Three pesticides significantly affected the pupae survival (females: *F*_6,42_ = 14.877, *P*<0.001; males: *F*_6,68_ = 14.877, *P*<0.001): 17.77±7.18% of females and 22.56±9.02% of males emerged from pupae treated with spinosad and 65±7.44% and 71.17±6.26% of females emerged from pupae treated with BCO and abamectin, respectively (Fig. 1c).

**Table 2:** Statistics from the GLM Multivariate analysis used to test the effects of insecticide, sex, residue age (1-h and 10-d) and of the interaction of the insecticide factor with all the other ones on the adult mortality (survival) and on the longevity of the surviving adults.

Test	Multivariate			Between-subjects					
Source of variation				Survival			Longevity		
	*df*	*F*	*P-value*	*df*	*F*	*P-value*	*df*	*F*	*P-value*
*Insecticide*	12	18.695	<0.001	6	91.371	<0.001	6	9.125	<0.001
*Sex*	2	1.116	<0.001	1	2.193	= 0.140	1	41.112	<0.001
*Residue age*	2	17.463	<0.001	1	14.27	<0.001	1	23.182	<0.001
*Insecticide* × *Sex*	12	5.133	<0.001	6	1.031	= 0.405	6	2.130	= 0.732
*Insecticide* × *Residue age*	12	2.416	= 0.006	6	3.467	= 0.003	6	2.943	= 0.039

### Sublethal effects

**Figure 2:**
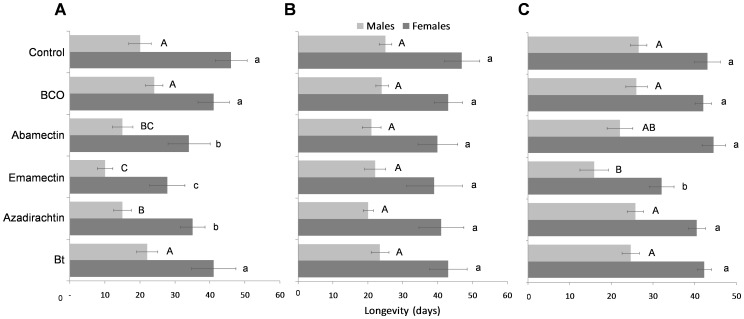
Sublethal effects on longevity. Means (± SEM) of longevity (days) of *Bracon nigricans* adults exposed to 1-h (A) or to 10-d pesticide residues (B) or of those emerged from treated cocooned pupae (C). For each figure, the bars followed by the same letter (lower case letters: female; upper case letters: male) are not significantly different (*P*>0.05; ANOVA with Tukey HSD post hoc test for multiple comparisons).

The tested biopesticides caused multiple sublethal effects on the surviving individuals in all the bioassays. The *insecticide* factor was significant for variation on longevity, amount of killed hosts, reproduction, and progeny sex-ratio (Tables 2 and 3). Adult longevity also varied depending on the pesticide residue age (significant *residue age* factor) and on its interaction with the tested insecticide (Table 2). Significantly lower longevity levels were found in the females (*F*_5,84_ = 34.523, *P*<0.001) and males (*F*_5,84_ = 15.241, *P*<0.001) that were exposed to 1-h old residues of azadirachtin, emamectin and abamectin (Fig. 2a). However, these effects were not consistent when the pesticide residues aged for 10 d, on females (*F*_5,84_ = 18.012, *P* = 0.081) and on males (*F*_5,84_ = 8.325, *P* = 0.152) (Fig. 2b). In the trial with pupae, only females (*F*_5,66_ = 39.067, *P*<0.001) and males (*F*_5,66_ = 17.201, *P*<0.001) emerged from the pupae treated with emamectin had a significantly shorter lifespan compared to those of the control (Fig. 2c).

**Table 4:** Reduction coefficient *E_x_* [48], IOBC toxicity classes [3], Doubling time (*DT*) and Intrinsic rate of increase (*r_m_*) [25] estimated for adults exposed to 1-h old and 10-d old pesticide residues, for adults emerged from topically treated cocooned pupae, and for the control population [33].

Active ingredient	Reduction coefficient (*E_x_*)			IOBC toxicity class			Doubling time (*DT*)			Intrinsic rate of increase (*r_m_*)		
	Adults		Pupae	Adults		Pupae	Adults		Pupae	Adults		Pupae
	1-h old residue	10-d old residue	Topical	1-h old residue	10-d old residue	Topical	1-h old residue	10-d old residue	Topical	1-h old residue	10-d old residue	Topical
Control	-	-	-	-	-	-	13.82	13.82	13.82	0.052	0.052	0.052
Abamectin	76.25	45.81	21.82	2	2	1	-	23.49	16.55	-	0.029	0.042
Azadirachtin	62.50	29.74	60.52	2	1	2	40.35	18.25	36.52	0.017	0.038	0.019
*Bacillus thuringiensis*	5.00	7.68	11.97	1	1	1	14.31	14.59	15.11	0.048	0.048	0.046
BCO	43.33	45.81	49.35	2	2	2	22.19	20.11	25.11	0.031	0.035	0.028
Emamectin benzoate	89.38	41.94	87.53	3	2	3	-	21.76	-	-	0.032	-
Spinosad*	100	100	80.6	4	4	3	-	-	-	-	-	-

*Calculated only for acute toxicity.

**Table:** Table 3. Statistics from the GLM Multivariate analysis used to test the effects of insecticide, residue age (1-h and 10-d), and of their interaction on the progeny production, progeny sex-ratio, and number of killed hosts.

Test	Multivariate			Between-subjects								
Source of variation				Progeny production			Progeny sex-ratio			Killed hosts		
	*df*	*F*	*P-value*	*df*	*F*	*P-value*	*df*	*F*	*P-value*	*df*	*F*	*P-value*
*Insecticide*	15	2.562	= 0.001	5	3.555	= 0.007	5	4.765	<0.001	5	7.241	<0.001
*Residue age*	3	7.116	<0.001	1	7.050	= 0.009	1	0.339	= 0.561	1	15.415	<0.001
*Insecticide* x *Residue age*	15	2.251	= 0.005	5	3.031	= 0.013	5	1.004	= 0.394	5	3.559	= 0.005

**Figure 3:**
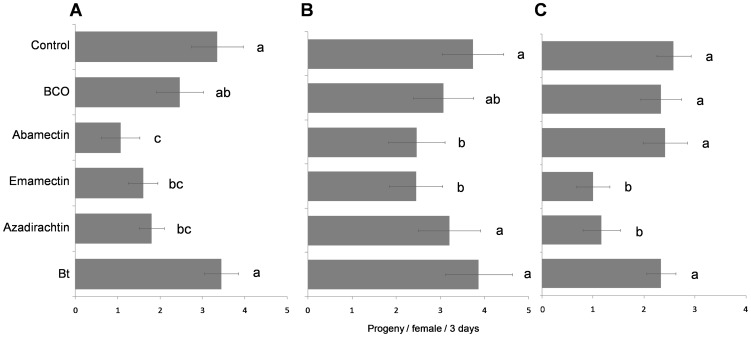
Sublethal effects on progeny production. Means (± SEM) of number of progeny produced in three d by each tested *Bracon nigricans* female exposed to 1-h (A) or to 10-d old pesticide residues (B) or by each emerged female from the treated cocooned pupae (C). For each figure, the bars followed by the same letter are not significantly different (*P*>0.05; ANOVA with Tukey HSD post hoc test for multiple comparisons).

In the trial with adults, progeny production, sex-ratio, and number of killed hosts varied significantly according to the applied insecticide (significant *insecticide* factor) (Table 3). Similar results, except for the progeny sex-ratio, were obtained when analyzing the effects of the *residue age* factor and its interaction with the *insecticide* factor (Table 3). Among the tested biopesticides, progeny production was significantly affected by emamectin in all three trials, i.e. 1-h (*F*_5,84_ = 6.607, *P*<0.001) and 10-d (*F*_5, 84_ = 3.845, *P* = 0.003) old residue, and in topical treatment of the cocooned pupae (*F*_5,66_ = 2.815, *P* = 0.001) (Fig. 3). Azadirachtin produced sublethal effects on pupae and on adults which were only exposed to 1-h old residues. Exposure to abamectin caused the lowest progeny production of the entire study (1.06±0.45 offspring per female in three d) in the 1-h old residue trial on adults (Fig. 3a). The progeny sex-ratio was significantly more male-biased only when the wasps were exposed to 1-h old residues of emamectin with 0.92±0.02 vs. 0.60±0.04 males/total progeny for the untreated control (*F*_5, 66_ = 0.632, *P* = 0.012) (data not shown).

**Figure 4:**
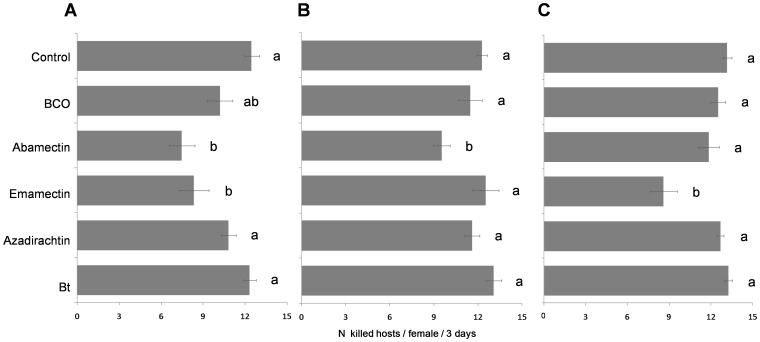
Sublethal effects on biocontrol activity. Means (± SEM) of number hosts killed in three d by one *Bracon nigricans* female exposed to 1-h (A) or to 10-d old pesticide residues (B) or by one emerged female from the treated pupae (C). For each figure, the bars followed by the same letter are not significantly different (*P*>0.05; ANOVA with Tukey HSD post hoc test for multiple comparisons).

During the three d of sublethal effect assessment, the females that were exposed to 1-h old residues of emamectin and abamectin killed significantly less host larvae compared to the untreated control, i.e. 35% and 44% less, respectively (*F*_5, 84_ = 12.180, *P*<0.001) (Fig. 4a). This effect persisted after 10 d only for abamectin (*F*_5, 84_ = 5.264, *P*<0.001; Fig. 4b), while emamectin significantly reduced the biocontrol services provided by the exposed females emerging from treated pupae (*F*_5,66_ = 7.895, *P*<0.001; Fig. 4c).

### Reduction coefficient (*E*_x_) and IOBC toxicity categories

Reduction coefficients *E_x_* were higher than 99% only after spinosad applications, and according to the IOBC toxicity categories, this insecticide was classified as harmful (class 4) for adults and moderately harmful (class 3) for pupae. Furthermore, its harmfulness remained high also for 10-d old pesticide residues (Table 4). When the lethal effect on female and the effects on production of female progeny were combined, we estimated an *E_x_* at 87.53% and at 89.38% (class 3) on pupae and on adults respectively, after the exposure to emamectin. *E_x_* decreased to 41.94% when females were exposed to 10-d old residues of emamectin. The same degrading trend was found for the other tested avermectin, abamectin, with an *E_x_* at 76.25% and at 45.81% (class 2); whereas, this insecticide was classified as harmless when applied to the pupae (Table 4). The botanical azadirachtin decreased in toxicity from slightly harmful (class 2) to harmless (class 1) when its residues were 10-d old, but showed a slightly harmful rating (*E_x_* at 60.52%) when applied to pupae. Class 2 was also attributed to BCO in all the bioassays, while the only tested biopesticide ranked as harmless (*E_x_*<30%; class 1) in all the bioassays was *Bt* (Table 4).

### Demographic growth parameters

All the tested biopesticides affected, *viz* decreased, all the estimated demographic indices (Table 4). In particular, 1-h old residues of emamectin and abamectin, and topical emamectin applied to cocooned pupae, caused very high decreases in the estimated population growth indices, resulting in wasp populations that could become extinct. In the 1-h old residue trial, Azadirachtin and BCO caused a delay of 25.4 and 8.4 d in population size doubling time (*DT*), respectively. When pupae were treated with azadirachtin, BCO, and abamectin, the delays in doubling time were 22.84, 11.29, and 2.73 d, respectively. However, the delay in the *DT* was lower when adults were exposed to 10-d old pesticide residues compared to the control population, resulting in DTs of 9.67, 7.94, 6.29, and 4.43 d for abamectin, emamectin, BCO, and azadirachtin, respectively. It was not possible to generate a demographic index for spinosad because all individuals died after residual contact, and mortality was very high after pupal exposure (Fig. 1). The effects of *Bt* on the population growth were minor since the estimated indices were very close to those of the control population. As a result the *DT* never exceeded 1.3 d (Table 4).

## Discussion

We used a new crop/pest/parasitoid system, i.e. tomato/*T. absoluta*/*B. nigricans*, to study how six biopesticides may affect population dynamics and the biocontrol activity of a generalist parasitoid. We showed that some of the tested biopesticides applied at the field concentrations caused a high degree of sublethal effects on various physiological and behavioral processes involved in the biocontrol activity, longevity, fertility, and in the progeny sex-ratio, despite low or no lethality. When integrating lethal and sublethal effects on female progeny production in the demographic model that includes the life history data specific for *B. nigricans* parasitizing the new exotic host *T. absoluta*, we highlighted that application of emamectin and abamectin may lead to a drastic decrease in population growth indices and the likelihood of the local extinction of the parasitoid population in the treated crops [24]. Spinosad has strong acute toxicity on parasitoids: they die rapidly owing to the exposure to the spinosad residues, even after exposure to 10-d old residues under greenhouse conditions. In contrast, *Bt* proved to be safe as it did not affect parasitism nor population growth.

### Differential effects among biopesticides, residues and tested instars

*Bracon nigricans* adults and pupae were very susceptible to spinosad (*E_x_*>99%, IOBC class 4). These results match those obtained on hymenopteran parasitoids and on predator arthropods through contact and ingestion assays in the laboratory [32]. Emamectin killed 17% and 47% of females and males, respectively, exposed to 1-h old residues. However, higher mortality rates were found in previous studies conducted exposing females of three other braconid species [*Cotesia vestalis* (Haliday), *C. plutellae* Kurdj and *Aphidius gifuensis* (Ashmead)] to dried residues of emamectin [52]–[54]. These differences could be due to the exposure substrate since overestimation of acute toxicity usually occurs when using pesticide residues on inert material, rather than on plant, such as in our study [11]. Emamectin acute toxicity on *B. nigricans* was not persistent until 10 d after the treatment. This pattern of toxicity degradation agrees with other studies conducted on other hymenopteran parasitoids and on an Anthocoridae predator species [48], [53]. By contrast, the individuals that survived this exposure and those that emerged from the pupae suffered severe sublethal effects. Emamectin sublethal effects were recorded in other studies on the development to adulthood of treated *Chrysoperla carnea* (Stephens) larvae [55] and of *Thricogramma* nr. *brassicae* larvae inside parasitized host eggs [56]. Interestingly, in this study this neurotoxic insecticide was, among the tested agrochemicals, the only one affecting the progeny sex-ratio and causing a significant increase in the proportion of males. Considering that arrenotokous parthenogenesis (i.e. unfertilized females produce only males) occurs in most braconid species [57], our results suggest that higher male progeny may be due to male sterility or mating behavior alterations. Although this aspect should be further investigated, behavioral alterations likely occur after wasps are exposed to neurotoxic insecticides [2], [14], [15].

Fresh residues of the other tested Avermectin, abamectin, were shown to be sublethal (i.e. caused no significant mortality of the females exposed as adults), but caused a high degree of sublethal effects on longevity, fertility, and biocontrol activity (the latter even after 10 d of the treatment). Severe sublethal effects on reproduction were also observed on heteropteran and Phytoseiid predator species [48], [58], [59]. Interestingly, we found lower levels of emergence from cocooned pupae after being sprayed with abamectin, while emerged adults did not show any sublethal effect. Our results on acute toxicity coincide only partially with those obtained by Bostanian and Akalach [20] that found high parasitoid mortality and a reduction of parasitization activity after exposing another braconid wasp, *Diaeretiella rapae* (McIntosh), to abamectin residues in glass vials. In contrast to our study which provided refuge for the wasps from the insecticide residue (i.e. untreated surface areas in the cup for retreat), their experimental setup did not allow the insects to avoid constant contact with the residues. In addition, as demonstrated by Iqbal et al. [60] on *Cotesia plutellae*, the residual activity of abamectin against parasitoid adults is markedly less on sprayed leaves compared to those residues on glass.

A similar trend was also observed with BCO which had no sublethal effects and whose acute toxicity was higher in adults exposed to1-h old residues rather than on sprayed pupae. In this case, the results can be attributed to the contact toxicity and repellent properties of plant essential oils [27], since citrus essential oil is the major component of BCO. Therefore, during the residual bioassay adults may have been repelled by the treated leaves and thus may have remained in less contact with the pesticide compared to the topically sprayed pupae. Interestingly, a recent study conducted spraying BCO on pupae of the mealybug parasitoid, *Anagyrus* sp. near *pseudococci* (Hymenoptera: Encyrtidae), showed no effects on the adult emergences from treated mealybug mummies [61]. These divergent results may be due to the differences in permeability between the mealybug mummy tissue and the *B. nigricans* silky cocoon.

Azadirachtin did not have a lethal impact on females exposed to 1-h old residue; however they suffered significant life history traits modifications that caused important delays in population growth. The properties of azadirachtin in reducing the fecundity and the life span of exposed adults are well recognized for arthropod pests (see Ascher [62] for a methodical review) and beneficials. Stark et al. [63] found that the reproduction of the braconid *Psytallia incisi* (Silvestri) parasitizing treated host larvae was significantly reduced; while in our study the females developed from sprayed pupae did not show any drop in reproduction. Stara et al. [64] proved that formulated azadirachtin (NeemAzal T/S) caused total mortality of the braconid *Aphidius colemani* Viereck after a 48-h exposure on glass plates, whereas when they tested the pure active ingredient it did not affect parasitoid survival and fecundity. However, although no mortality was observed after exposing three heteropteran predators to sprayed leaves, their fertility was significantly affected [48], [65].

As already proved for two predator species [8], [48], [66], *Bt* proved to be totally harmless in all the trials (both in terms of lethal and sublethal effects). This is likely due to the failure of the *Bt* toxins to reach the parasitoid gut, and/or the inability of the toxins to bind to the mid-gut receptors [67]. In addition, our results suggest that the residues of the adjuvant compounds present in the *Bt* formulations did not impact the parasitoid.

### Importance of results for IPM and organic farming in tomato

Our study provides information that could be useful for IPM and organic programs for identifying harmful biopesticides. Such products may compromise the efficacy of IPM programs, in particular pest control on treated crops, by preventing the efficient colonization or re-colonization of parasitoid populations. This is particularly true when the pesticides are highly persistent, such as in the cases of spinosad and of the two avermectins. These results imply that agrochemicals classified as biopesticides can be of major concern when they are supposed to be integrated with the ecosystem services spontaneously or artificially provided by natural enemies. Therefore, given the results, the combined use of biopesticides and natural enemies, specifically braconid wasps, should not be considered for effective and sustainable IPM and organic programs. Although the two avermectin-based biopesticides are effective in lepidopteran pest control [47], they should not be applied in cropping systems where sustainable efficacy of natural enemies against pests is a key component. Unfortunately, a similar conclusion applies to spinosad that is currently authorized and recommended for organic cropping systems.

These present results corroborated with those obtained in other studies on predators suggest that other organic certified pesticides with higher selectivity would be preferable [32], [35]. For example, since azadirachtin and BCO had small side effects on adults, were not persistent in tomato plants, and they affected treated pupae, these products should be applied during the period of low presence of parasitoid pupae on the crop, i.e. before the parasitoid arrival into the crop. Overall, among the tested products, the safest insecticides for *B. nigricans* appears to be *Bt*. Thus when dealing with lepidopteran pests, this biopesticide should be preferred because: (i) it proved safe for *B. nigricans*, despite testing in at one of highest recommended rates both in terms of toxin international units (9000 I.U. µg^−1^) and in recommended concentration (200 g hl^−1^); (ii) it has been shown to not interfere with predator establishment in tomato [8], [66] and (iii) it is highly efficient even against lepidopteran pests in tomato [68].

### Implications of the approach for pesticide risk assessment

This study clearly demonstrates how the tested pesticides induce more subtle rather than acute affects. As a matter of fact, all of the investigated physiological and/or behavioral traits are useful in order to better assess the actual impact of the tested biopesticides on the parasitoids population dynamics, as well as on the biocontrol services they may provide after the pesticide application. This is particularly true in the case of the Avermectins since the survival rate of the females exposed to their 1-h old residues was reduced by a maximum of 15%, while longevity, fertility, and biocontrol activity decreased from 25% to 80%. Furthermore, the females that survived to the emamectin exposure had a progeny sex-ratio strongly male biased (>0.9 mm/tot). Consequently, from an ecotoxicological perspective, this study provides a basis for further research aimed at assessing the behavioral and physiological mechanisms that have caused the progeny sex-ratio disorder, and reduction of fertility and biological control activity.

Even if the demographic approach has not been widely adopted up until now, this method, as demonstrated in this study, provides more information about pesticide effects on population dynamics than the traditional risk assessment. There are clearly great advantages in using demography for the estimation of pesticide impacts on non-target organisms. This approach incorporates species specific life history data, and both lethal and sublethal effects on the reproduction, into a single dimensionless number which is easy to compare [25]. Furthermore, we believe that the toxicity of abamectin and emamectin (ranked as slightly harmful and moderately harmful following the IOBC categories) on *B. nigricans* correspond to the cases in which the wasp populations go into local extinction [24]. Thus, the demographic approach is indeed much more accurate than lethal concentration estimates or, as proved in this study, those advocated by the IOBC classification.

With regard to any reductions in fertility (namely *E_fx_*), our experimental model assumes that the reductions in fertility occur throughout the female's life, although we only assessed this trait during the three d after the exposure. Indeed, it is speculated that some insects, mainly insecticide-resistant species/strains, need time after the exposure to detoxify the toxicant metabolically [69]. Thus, our model does not allow assessing whether exposed females can recover and have higher reproductive outputs later in their lifespan.

## Conclusion

The major purpose of sustainable crop protection strategies is to preserve and/or increase the natural mortality factors of the pests by combining synergistic control measures. Better knowledge on risks associated with specific pesticide use towards natural enemies is of primary importance when incorporating them into IPM and organic programs. The results of this study show that side effects of pesticides can vary largely depending upon various factors considered, such as endpoint (lethal vs. sublethal and instar tested), pesticide persistence, and developmental strategies of the non-target species in question. Consequently, comprehensive and specific risk assessment should be conducted before incorporating pesticides into IPM and organic programs, especially in the case of newly developed insecticides that are often *slower acting* and may cause multiple chronic sublethal effects rather than just immediate acute mortality.

These results were obtained under laboratory conditions in a high exposure scenario, thus semi-field and field experiments should also be carried out. Nevertheless, risk prediction models from laboratory data are necessary for the rational selection of the insecticides to be employed in successful IPM and organic control program against new invasive pests (such as *T. absoluta*) or when new pesticides are available, such as in the case of new biopesticides. Our findings demonstrate how population models can be used in developing more ecologically relevant population-level endpoints, and how sublethal effects can also impair the ecosystems services provided by parasitoids that survive pesticide exposure but that are affected due to sublethal effects.
